# Non-rigid image registration to reduce beam-induced blurring of cryo-electron microscopy images

**DOI:** 10.1107/S0909049512044408

**Published:** 2012-11-29

**Authors:** Fatemeh Karimi Nejadasl, Manikandan Karuppasamy, Emily R. Newman, John E. McGeehan, Raimond B. G. Ravelli

**Affiliations:** a Department of Molecular Cell Biology, Leiden University Medical Center, PO Box 9600, 2300RC Leiden, The Netherlands; b Biophysics Laboratories, University of Portsmouth, Portsmouth PO1 2DY, UK

**Keywords:** cryo-electron microscopy, single particle, beam-induced movements, exposure series, radiation damage

## Abstract

The typical dose used to record cryo-electron microscopy images from vitrified biological specimens is so high that radiation-induced structural alterations are bound to occur during data acquisition. Integration of all scattered electrons into one image can lead to significant blurring, particularly if the data are collected from an unsupported thin layer of ice suspended over the holes of a support film. Here, the dose has been fractioned and exposure series have been acquired in order to study beam-induced specimen movements under low dose conditions, prior to bubbling. Gold particles were added to the protein sample as fiducial markers. These were automatically localized and tracked throughout the exposure series and showed correlated motions within small patches, with larger amplitudes of motion vectors at the start of a series compared with the end of each series. A non-rigid scheme was used to register all images within each exposure series, using natural neighbor interpolation with the gold particles as anchor points. The procedure increases the contrast and resolution of the examined macromolecules.

## Abstract 2

Cryo-electron microscopy images of vitrified large macromolecular complexes can become blurred due to beam-induced specimen alterations. Exposure series are examined, and rigid and non-rigid image registration schemes are applied to reduce such blurring.

## 1. Introduction

Cryo-electron microscopy (cryo-EM) is a technique that can be used to obtain three-dimensional structures of large macromolecular complexes. High-resolution structures have been obtained by averaging numerous identical copies of the investigated complex, either in crystalline, helical, polymerized or isolated form (Orlova & Saibil, 2011 ▶). The latter technique is referred to as single-particle cryo-EM. The typical dose used to record a single-particle cryo-EM image is of the same order of magnitude as the tolerable dose limit for a macromolecular crystal before it loses half of its X-ray diffraction intensity. For example, it was estimated that an integrated incident flux of 5 e^−^ Å^−2^ of 120 keV electrons for a 150 nm thin vitrified *Lumbricus terrestris* hemoglobin sample resulted in a deposited dose of 5.5 MGy (Karuppasamy *et al.*, 2011 ▶). Cryo-EM images used for single-particle reconstructions are typically recorded with 5–20 e^−^ Å^−2^ (most commonly, these numbers are referred to as ‘electron dose’). The experimental dose limit in X-ray crystallography has been reported to be 30 MGy (Owen *et al.*, 2006 ▶). A typical integration time in cryo-EM is 1 s, whereas the dose limit in crystallography is reached in minutes on most third-generation undulator beamlines: the typical time required to deposit, for example, 10 MGy varies by at least two orders of magnitude between the two techniques. Systematic radiation damage studies in X-ray crystallography revealed some very generic consequences of the interaction between the ionizing radiation and the vitrified macromolecular specimen: molecules undergo small rotations and translations, the unit cell expands, *B*-factors increase, disulfide bonds break, metals become reduced and acidic residues become decarboxylated (Burmeister, 2000 ▶; Ravelli & McSweeney, 2000 ▶; Weik *et al.*, 2000 ▶). Radiolysis of the vitrified water, in addition to the biomolecules themselves, results in the formation of hydrogen gas (Meents *et al.*, 2010 ▶). For cryo-EM, observed radiation damage effects include the rotation and translation of molecules (Brilot *et al.*, 2012 ▶) as well as hydrogen gas formation (Leapman & Sun, 1995 ▶).

The large amount of energy that is deposited in a matter of a few seconds by the high-energy electron beam interacting with the thin vitreous sample will leave an imprint on the cryo-EM specimen. Single-particle cryo-EM studies commonly make use of holey support grids in which the holes are filled by the vitrified specimen. The absence of a support layer is favorable in terms of background and it reduces the risk of inducing preferred particle orientation. However, as a consequence, the unsupported vitrified ice layer is fragile, non-rigid, and can build up charge as well as mechanical stress. Charging, mechanical stress and differential expansion of the vitrified specimen *versus* the support film lead to apparent beam-induced movements, either by beam deflection, real physical sample movement, or a combination of both. Attempts to reduce beam-induced movements include pre-illuminating the support grid overnight with the electron beam (Ge & Zhou, 2011 ▶), the use of a thicker carbon support to increase the conductivity, coating the holey support films with a metallic titanium/silicon film (Glaeser & Downing, 2004 ▶), or the use of doped silicon carbide (Yoshioka *et al.*, 2010 ▶). Varying parameters such as dose rate (Chen *et al.*, 2008 ▶; Karuppasamy *et al.*, 2011 ▶; Brilot *et al.*, 2012 ▶), aperture size, beam conditions and support-film hole size (Brilot *et al.*, 2012 ▶) seem to influence the amount of beam-induced movements. However, in the absence of a robust support layer there is no guarantee of complete motion removal.

Recently, it was shown that large 70 MDa rotavirus particles can be used as an effective probe to monitor and quantify beam-induced motions (Brilot *et al.*, 2012 ▶). A drum-like motion of the whole ice layer was proposed, and rotations as large as a few degrees were observed for individual particles. The size and regularity of these viral particles allowed for the accurate determination of the translation and orientation of each individual particle, within 0.2 Å and 0.2° (Brilot *et al.*, 2012 ▶).

We wished to determine the three-dimensional structure of a ∼700 kDa heterogeneous complex comprising the nucleosome assembly protein (NAP) and histones (Newman *et al.*, 2012 ▶). Individual images recorded with 10–20 e^−^ Å^−2^ in general showed poor contrast whereas images recorded with fewer electrons were sharper but noisy. We set up to collect exposure series (stroboscopic data collection; Henderson & Glaeser, 1985 ▶; Typke *et al.*, 2007 ▶; Karimi Nejadasl *et al.*, 2011 ▶; Karuppasamy *et al.*, 2011 ▶) with different defoci, under illumination conditions prior to which gas bubbling occurred. Gold fiducial markers were added to aid image registration. Beam-induced movements were still observed after removing the global shift between images and averaging them. Even after compensation for in-plane rotations, scale and shearing, beam-induced motions remained.

In this paper we describe the use of non-rigid image registration to correct for specimen deformations induced by the electron beam. For this purpose we detected and tracked gold fiducial markers in each image sequence. Tracking errors were removed and, as a result, the gold particles of each candidate image could be connected to the reference image. The motion vectors of the gold fiducial markers were subsequently used as anchor points. Motion vectors of other pixels were obtained from natural neighbor interpolation of gold particle motion vectors. The transformed candidate image was obtained by applying these motion vectors on the candidate image. This procedure was repeated for all candidate images within an image sequence, after which beam-induced motion-corrected averages could be calculated. It is shown that protein particles show a better contrast and sharper averages after correction of beam-induced movements derived from neighboring gold particles.

## 2. Methods

### 2.1. Experimental methods

#### 2.1.1. Sample preparation

Purified NAP_2_.H3/H4 histone chaperone protein complex from *Xenopus laevis* was used as a test sample for this study (Newman *et al.*, 2012 ▶). A gradient fixation protocol (Grafix; Kastner *et al.*, 2008 ▶; Stark, 2010 ▶) was applied to chemically stabilize the sample and to improve the sample quality of the inherently heterogeneous complex. The protein complex was used at a concentration of 0.75 mg ml^−1^ in a buffer consisting of 20 m*M* Tris pH 7.2, 3 m*M* MgCl_2_ and 150 m*M* NaCl. Fifteen microliters of sample was mixed with 1.2 µl of 5 nm proteinA gold (CMC-UMC, Utrecht, The Netherlands) as fiducial marker. Three and a half microliters of the resulting sample was applied to 200 mesh glow discharged C-flat 2/2-type copper grids (2 µm hole size, extra thick carbon; ProtoChips Inc). The blotting was done inside an FEI Vitrobot Mark IV using a 3 s blotting time with 100% relative humidity at room temperature. Subsequently, the blotted grid was vitrified in a cold (77 K) liquid mixture of ethane and propane. The dilution of the stock solution of 5 nm proteinA gold particles was optimized such that the vitreous sample spanning a 2 µm carbon hole would typically contain between 10 and 100 gold fiducial markers.

#### 2.1.2. Data collection

We collected exposure series (stroboscopic data collection; Typke *et al.*, 2007 ▶; Karimi Nejadasl *et al.*, 2011 ▶; Karuppasamy *et al.*, 2011 ▶) with an incident flux density of 5 e^−^ Å^−2^ s^−1^ and an integration time of 1 s per image. Series of 13 CCD images were collected from each position at two different requested defoci (first ten images at defocus −1 µm followed by three images at defocus −6 µm). All data were recorded on a FEI Tecnai F20 transmission electron microscope (FEI Company, Eindhoven, The Netherlands) equipped with a field emission gun operating at 200 kV without using an energy filter. Images were collected as fast as possible after each other (Faas *et al.*, 2012 ▶), resulting in, on average, 13 images per minute. The magnification at the detector plane was 65500×. The images were recorded on a 4k × 4k Gatan UltraScan 4000 slow-scan CCD (Gatan Inc., Pleasanton, USA) on-axis camera and hardware binned providing 2k × 2k images with a pixel size of 4.6 Å × 4.6 Å square. Other microscope parameters were: chromatic aberration, 2 mm; spherical aberration, 2 mm; energy spread, 0.7 eV; illumination aperture, 0.1 mrad; condenser aperture number, 3 (size of 100 µm); objective aperture, 3 (70 µm); and spot size index, 6. A total of 45 series were collected in one microscope session and further analyzed.

### 2.2. Computational methods

#### 2.2.1. Detection of fiducial gold particles

Individual images were corrected for statistical outliers (Vulovic *et al.*, 2010 ▶). Sample drift was accounted for by translational alignment of all images within each series by cross correlation, using the first image as a reference. Translation vectors were calculated with sub-pixel accuracy. The alignment was carried out by applying a corresponding phase shift in Fourier space.

The positions of the 5 nm fiducial gold particles were detected as described previously (Karuppasamy *et al.*, 2011 ▶). Briefly, images were Laplacian of Gaussian filtered for a range of σ values to account for the observed variation in gold particle size. The resulting images were added together. The center of the brightest regions within this sum formed a starting set for the gold particle positions. These coordinates were used as input for the subsequent step, particle tracking.

#### 2.2.2. Gold particle tracking

The analysis of the motion of the individual gold particles within a series depends on a successful gold particle tracking procedure. We exploited the experimental constraints of our data: within a field of view of 1 µm^2^ we typically observed tens of gold particles. At this magnification and dilution, neighboring gold particles appeared to be moving in a similar direction and magnitude. The individual particles moved overall net in one direction as a function of dose. The magnitude of the movement between successive frames was similar and slowly decreased with dose. The maximum direction and magnitude differences of motion vectors between successive frames were, respectively, 45° and 5 pixels. We observed both constraints to be true for doses prior to which bubbling occurred. These spatial and temporal constraints were used to ensure reliable and robust particle tracking.

The gold particles were first tracked based on temporal constraints. Next, the spatial constraints were employed to remove wrongly tracked particles. The combined use of these constraints resulted in a reliable gold particle tracking procedure.

(i) *Tracking.* The first image was selected as a reference image. The procedure was repeated in the forward direction, although, in principle, it could also be applied in the reverse direction.

The tracking was initialized with the gold particle coordinates as determined for the reference frame. These particles were matched to the particle coordinates as determined for the subsequent frame, based on distance. The nearest distance was used to match gold particles among successive frames. Only distances smaller than a threshold of 30 pixels were accepted: the tracking was marked as failed if no pair could be found that was separated by a distance smaller than the threshold. The scheme was repeated for all frames within a series by cumulatively adding the displacements to the current gold locations, resulting in a tracking list for each point of the reference image. The list could include flags for failed tracking events.

(ii) *Removal of tracking errors.* Each tracking list could be contaminated both by failed tracking events and wrongly tracked points. The tracking lists with too many failed tracking events were discarded. For each pair of images, wrongly tracked points were identified exploiting the observation that neighboring gold particles move in similar directions with similar magnitudes. The set of gold particles that were localized on both images were systematically analyzed in groups of three neighboring particles. A region containing these three neighboring particles was selected from the reference image. This region was shifted three times, according to each of the three motion vectors of the individual particles, and a cross-correlation was calculated for each of these shifts. The shift that resulted in the highest cross-correlation was selected as the best displacement. The other shifts were compared with this best motion vector. Shifts that deviated too much (more than ten pixels in distance and 30° in direction) from the best motion vector were rejected, and their corresponding gold particles were marked as wrongly tracked. For our data, it was not necessary to re-track the wrongly tracked gold particles as there were enough remaining fiducial markers to proceed with the subsequent steps.

The procedure of tracking error removal was repeated for each image within the series, always using the first image as a reference image.

#### 2.2.3. Rigid registration

Different rigid models were tested for describing the observed movements of the tracked fiducial markers. The root-mean-square difference between the predicted and observed positions was used as a metric to evaluate the different rigid models. We compared three different in-plane rigid-transformation models: solely translations, similarity (translations, in-plane rotation and scale) and homography (translations, in-plane rotation, scale, scale ratio, shearing and two parameters for perspective projection; Hartley & Zisserman, 2004 ▶). These models contain, respectively, 2, 4 and 8 free parameters.

#### 2.2.4. Non-rigid image registration

Rigid models could not accurately model all of the observed beam-induced fiducial marker movements. We therefore examined non-rigid image registration protocols for removing the geometrical deformations that were left after correction of the global deformation. The gold fiducial markers were used as anchor points: their motion vectors are known from the tracking procedure. These were used to obtain motion vectors for arbitrary pixel points by natural neighbor interpolation (Sibson, 1981 ▶; Okabe *et al.*, 2000 ▶).

Natural neighbor interpolation works as follows. Suppose there are a set of points in a plane, in this case the gold fiducial markers. These points can be used to construct a Voronoi diagram (Okabe *et al.*, 2000 ▶) which decomposes the plane into regions, named Voronoi cells. All points within one Voronoi cell have a distance to its fiducial marker that is smaller than the distance of that point to all other fiducial markers. Now suppose that a new fiducial point is added to an existing Voronoi diagram. Such a point will create one new Voronoi cell which will intersect a certain number of old Voronoi cells. The points corresponding to these old Voronoi cells are defined as the natural neighbors of the newly inserted point.

Suppose, for example, that a pixel has six natural neighbors among the fiducial markers. For each of these six fiducial marks a motion vector is given. The estimated motion for this pixel is now the weighted sum of these six motion vectors. There are different ways to determine such weights (Farin, 1990 ▶; Sugihara, 1999 ▶; Hiyoshi & Sugihara, 2004 ▶; Bobach *et al.*, 2006 ▶), and the following was used here: if the pixel were added to the Voronoi diagram of fiducial marks, its new Voronoi cell would occupy a certain area and this new Voronoi cell by definition would exactly intersect the old Voronoi cells of the natural neighbors. For each of the natural neighbors the ratio between the intersection of the new Voronoi cell of the particular pixel and the old Voronoi cell of the fiducial mark can be determined. These ratios are then used to define the weight over the natural neighbors.

After modeling the motion vectors for all pixels, the second image within a series is nearest neighbor interpolated for the new locations. The above-mentioned procedure is repeated for all images except for the first one which was used as a reference. The resulting transformed image series can subsequently be averaged.

## 3. Results

### 3.1. Rigid registration

Each series of images was recorded at two different defoci (−1 µm and −6 µm requested defocus). The two-condenser system of the F20 Tecnai microscope does not allow for adjustable parallel illumination: the second condenser lens was tuned yielding the correct incident flux density and beam size, thereby compromising the beam parallelism. Consequently, the image could show rotational and magnification changes upon altering the defocus. This phenomenon was explored to test the rigid registration model.

**Figure 1:**
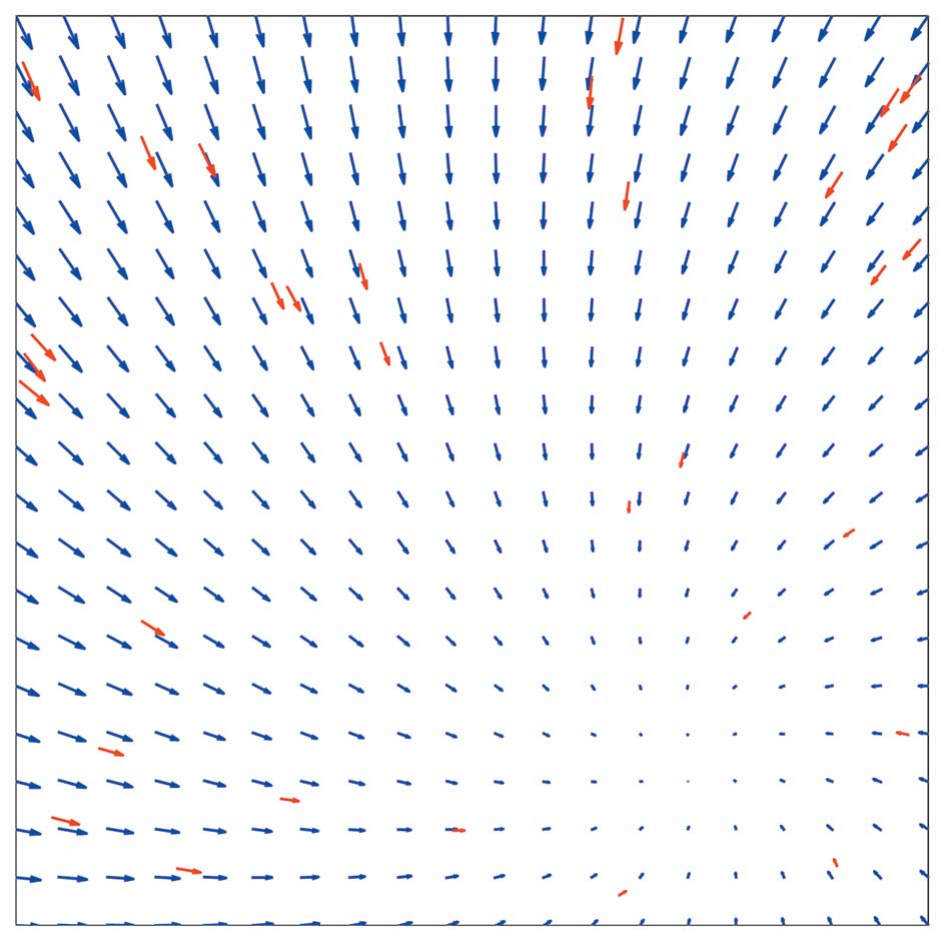
Rigid movement upon defocus change. The average of ten images collected at −1 µm defocus was compared with the average of three images collected at −6 µm. The motion vectors of the tracked gold particles, magnified five times, are shown in red. The blue vectors show the displacement landscape (magnified five times) of the whole image.

Fig. 1 ▶ shows the result of the registration of ten averaged images recorded at −1 µm defocus *versus* three averaged images recorded at −6 µm defocus. Images recorded with two different defocus values are known as focal pairs. In red, the motion vectors between the gold particles of both averaged images are shown. The motion vector landscape (displacement landscape) between the two averaged images is shown by blue vectors (Fig. 1 ▶). The resulting root-mean-square errors (RMSE) between the measured and observed gold particle positions between focal pairs were typically, after rigid registration, smaller than one pixel. The gold particle coordinates were used to determine the scale and rotation parameters for each defocus pair. These values were, averaged for the 45 series, 1.0086 (0.0004) and 0.149° (0.012), respectively.

**Figure 2:**
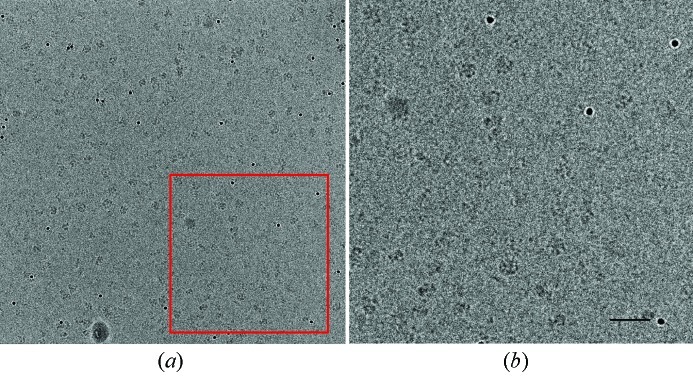
Averaged image of a series recorded at two different defoci. The images were registered with a rigid movement (see Fig. 1 ▶). Panel (*b*) is a magnification of the area marked by a red box in panel (*a*). The scale bar shown in (*b*) corresponds to 50 nm.

The two averaged images could be combined after applying the rigid movement to one of them. Fig. 2 ▶ shows the resulting average of all 13 original images, recorded at different defoci. The gold particles are well defined and spherical and clear protein particles can be seen. The figure shown does not include a correction for the focus-dependent contrast transfer function, as can be recognized from the white halo around the gold particles which becomes apparent in the higher defocus images. Such averages were used for particle picking, and were therefore mainly concerned with the geometric transformation between the close-to-focus and out-of-focus frames. For this purpose, images like those shown in Fig. 2 ▶ are perfectly adequate.

For our data collection scheme, rigid registration worked well for focal pair alignment.

### 3.2. Rigid registration cannot model all beam-induced movements

The series used in Figs. 1 ▶ and 2 ▶ is not representative for all the images series which were collected: the RMSE of the tracked gold particle positions between the first and the tenth frame of that series (all collected at the same defocus) is less than a few pixels (∼10–20 Å).

**Figure 3:**
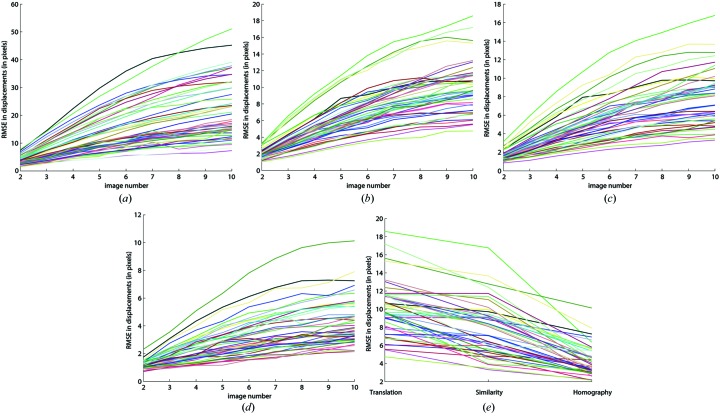
Root-mean-square error (RMSE) between predicted and measured gold particle positions using different similarity models. A total of 45 series were collected: for each series the RMSE is shown with a differently colored line as a function of frame number. Panel (*a*) shows the original RMSE, without registration. A drift correction was applied for the data shown in panel (*b*). Panel (*c*) shows a rigid movement registration in which translation, rotation and scale were refined, whereas panel (*d*) shows a full rigid movement registration where eight parameters were refined. The RMSE as a function of registration model is shown in panel (*e*).

Fig. 3 ▶ shows graphs of the RMSE as a function of frame number for all the recorded image series. Fig. 3(*a*) ▶ shows the RMSE of the original data, in absence of any alignments. All lines are monotonic, and the slope becomes less for higher frame numbers. The poor numbers shown in Fig. 3(*a*) ▶ (some RMSE are as large as 50 pixels) improve substantially after drift-correction. The first ten images were collected in less than 1 min and the median sample drift within this period was 7 nm. Correction for the drift produces RMSE values between frame 1 and 10 that vary between 4 and 18 pixels (Fig. 3*b* ▶). All lines are monotonic with decreasing slope for higher frame numbers as was observed in Fig. 3(*a*) ▶. The RMSE values improve further by increasing the number of parameters that can be refined for different rigid registration models [Fig. 3(*c*)–3(*e*) ▶]. However, even for the homography model, significant errors remain (RMSE between frame 1 and 10 varied between 1 and 10 pixels).

**Figure 4:**
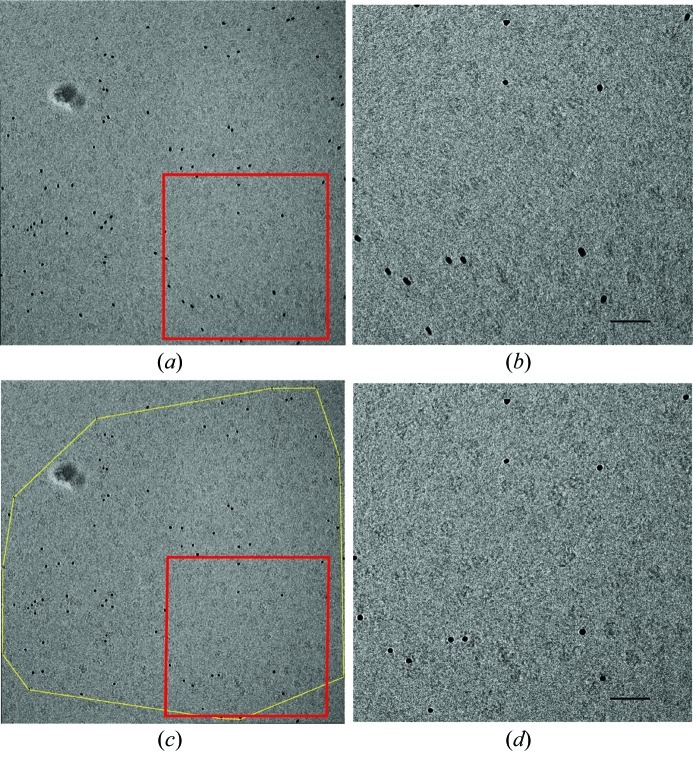
Rigid registration of a series of ten images collected at −1 µm defocus. The resulting image is blurred as not all beam-induced movements could be accounted for. Panel (*b*) is an enlargement of the area marked by the red box in panel (*a*). Panels (*c*) and (*d*) [enlargement of (*c*)] show the non-rigid registration of the same series. The yellow contour in panel (*c*) shows the region of the image that was corrected. In contrast to (*a*) and (*b*), gold particles have become sharp and more protein particles become visible in (*c*) and (*d*). The scale bar shown in panels (*b*) and (*d*) corresponds to 50 nm.

Fig. 4 ▶ shows a tenfold-averaged image after homographic registration. The RMSE of the tracked gold particle positions between frame 1 and 10 is 24.2, 9.4, 9.1 and 5.4 pixels for original, translational, similarity and homographic registration, respectively. The magnified view of the averaged image (Fig. 4*b* ▶) clearly shows residual alignment errors, which could not be modeled by the rigid registration models. The gold particles are smeared, and very few protein particles could be identified within the blurred image. The exposure of the specimen to the electron beam induced apparent movements that cannot be modeled by rigid registration schemes.

### 3.3. Non-rigid registration

**Figure 5:**
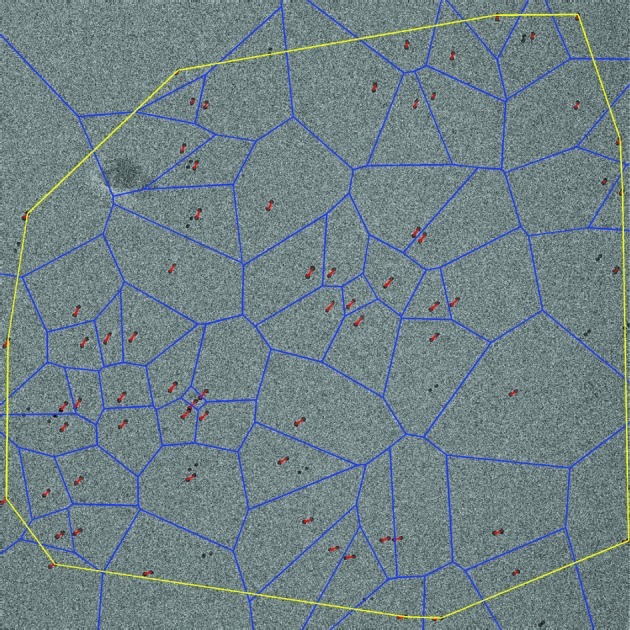
Voronoi diagram used for non-rigid registration. The first and last image within a series of ten images were averaged. The motion vectors between tracked gold particles are shown as red vectors. The Voronoi diagram is shown in blue. This diagram is used to non-rigidly register the region within the yellow outline.

**Figure 6:**
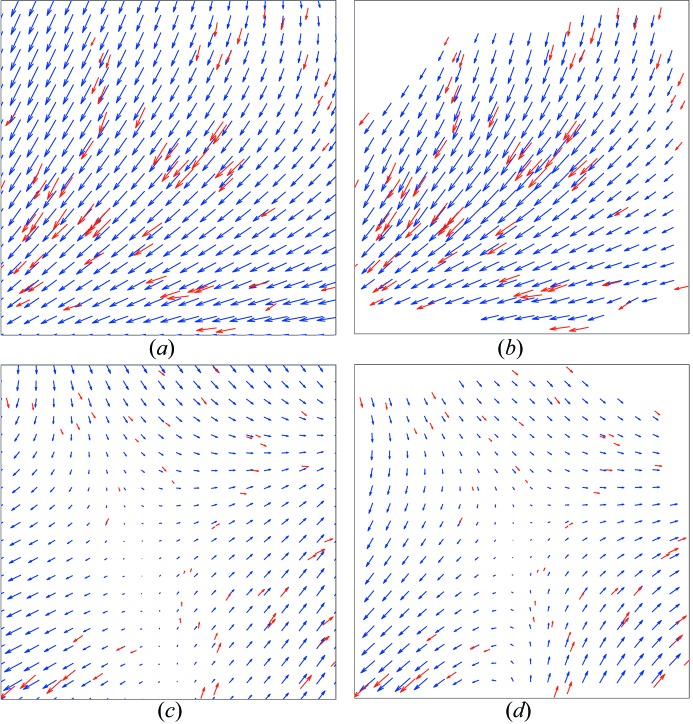
Comparison of a homography model and non-rigid registration to correct for beam-induced movements. Two different series are shown. Panels (*a*) and (*b*) show in red the motion vectors of gold particles tracked between the first and the tenth image for the series that was also used in Figs. 4 ▶, 5 ▶ and 7 ▶. All vectors are magnified five times. Panel (*a*) shows in blue the displacement landscape employing a rigid homography model. This model cannot accurately describe all the observed motions (*e.g.* upper left corner); the resulting RMSE between the observed and modeled gold particle positions is 5.4 pixels. Panel (*b*) shows, in blue, the displacement landscape for the non-rigid registration. The empty white areas in the corners represent those pixels for which no correction could be made (see also Fig. 5 ▶). Panels (*c*) and (*d*) show the displacement landscape for another series. Panel (*c*) displays the homography model (RMSE is 4.4 pixels); the non-rigid registration is shown in panel (*d*).

The tracked gold particles within the image shown in Fig. 4(*a*) ▶ were used to construct a Voronoi diagram. The resulting diagram is shown in Fig. 5 ▶: individual Voronoi cells are shown in blue whereas the motion vectors of the tracked gold particles are shown in red. Such diagrams were used for natural neighbor interpolation as described in §2.2.4. Fig. 6(*b*) ▶ shows the resulting displacement landscape for the tenth image of the same series that was used for Figs. 4 ▶ and 5 ▶. As a comparison, the displacement landscape has also been calculated for the rigid homography model (Fig. 6*a* ▶). Differences are particularly noticeable in the upper and lower right corners of the image: the non-rigid registration model provides more flexibility for dealing with local deformations. The displacement landscape, however, remains relatively smooth, imposed by the correlated motion of neighboring fiducial markers.

Fig. 6(*b*) ▶ is representative for all of the image series that required non-rigid registration. Another example is shown in Figs. 6(*c*) and 6(*d*) ▶, where panel (*c*) shows the displacement landscape using the rigid homography model and panel (*d*) shows the displacement landscape corresponding to the non-rigid registration. Again, the gold particles seem to move in patches and neighboring fiducial markers show correlated motions.

The frames numbered 2 to 10 were transformed according to each of the corresponding non-rigid registrations, after which all ten frames could be averaged. The resulting image is shown in Figs. 4(*c*) and 4(*d*) ▶. All gold particles are now, unlike in Figs. 4(*a*) and 4(*b*) ▶, well defined and sharp; the averaged image is no longer blurred, and many individual protein particles become apparent.

### 3.4. Reduction of image blurring

Blurring originating from beam-induced specimen movements can be reduced by the collection of exposure series and the application of non-rigid registration schemes. The question of whether this would also improve single-particle reconstructions is not answered in this work: the data presented here were collected from a specimen of unknown structure. The NAP_2_·H3/H4 sample is known to be heterogeneous, as reported recently (Newman *et al.*, 2012 ▶). Unlike Brilot *et al.* (2012 ▶), who could analyze beam-induced rotation and translation of 70 MDa rotavirus double-layer particles, no accurate three-dimensional models of the NAP-histone oligomers are so far available.

**Figure 7:**
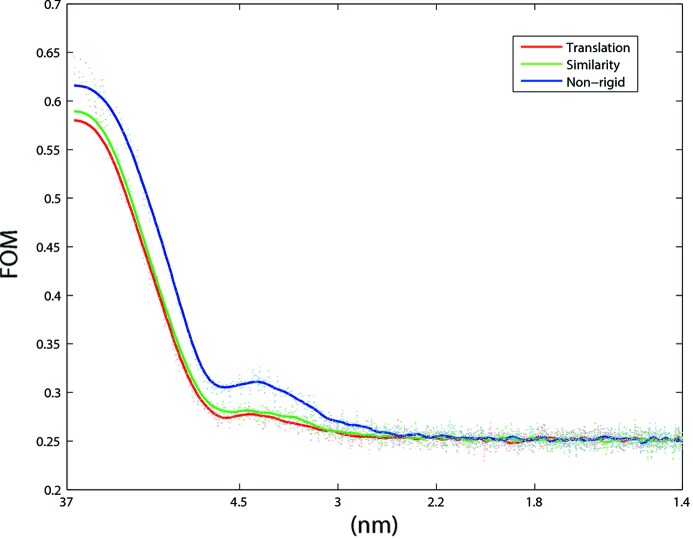
Figure of merit *versus* resolution for different registration schemes applied to the image series shown in Figs. 4 ▶ and 5 ▶. The figure of merit (FOM) was calculated as described by Karimi Nejadasl *et al.* (2011 ▶) for the first ten images within the series. A clear improvement in the signal is observed upon non-rigid registration.

A statistical metric was employed to evaluate the effect of different registration schemes. A figure of merit (FOM), the cosine of phase errors of an exposure series (Karimi Nejadasl *et al.*, 2011 ▶), was calculated from the first ten images within each series, and plotted as a function of resolution. Larger FOM values correspond to more consistent data. Fig. 7 ▶ shows the FOM graphs for different registration schemes employed on the series used in Figs. 4 ▶–6 ▶ ▶. The non-rigid registration scheme gives a significant improvement of the signal compared with all rigid models, over almost the entire resolution range. Part of this amelioration originates from the improved alignment of the fiducial markers themselves.

## 4. Discussion and conclusions

Radiation damage is not a binary nuisance. Single-particle cryo-EM studies generally make use of data that were collected with less than a certain number of incident electrons per squared Ångström (typically <20 e^−^ Å^−2^). Such a limit does not preclude the presence of radiation damage: comparable doses (in Grays; Karuppasamy *et al.*, 2011 ▶) used in X-ray crystallography are known to induce global as well as specific alterations within the macromolecule (reviewed by Ravelli & Garman, 2006 ▶). The use of exposure series provides the data to devise more sophisticated processing schemes in which the effects of radiation damage in single-particle cryo-EM can be studied and, ultimately, accounted for, mitigated or even exploited.

The exposure series presented here were all collected using a CCD camera. Previously, we presented a toolbox to characterize CCD cameras for transmission electron microscopy (Vulovic *et al.*, 2010 ▶). The camera used for the data presented here has a readout noise of 3.5 ADU, a conversion factor of 23 ADU per primary electron, and a readout time of >8 s for unbinned images. The collection of exposure series will become much more favorable for direct electron detectors which are typically composed of a series of short-exposure frames. The readout noise is significantly reduced and full-frame readout speed can be 40 Hz (Brilot *et al.*, 2012 ▶) or higher. The spectacular recent progress in detector hardware technology makes the call to account for radiation damage timely.

Radiation-damage-induced movements are amplified for biological material embedded in a thin layer of vitreous ice that spans the holes of a support film (Brilot *et al.*, 2012 ▶). Recently, it was shown that the use of a strong and conductive support significantly reduced beam-induced motions of two-dimensional paraffin crystals and yielded better contrast compared with the collection of exposure series with a zero-noise-readout Medipix II detector (Glaeser *et al.*, 2011 ▶). Unfortunately, ice-embedded biomolecular structures are best imaged in the absence of a strong conductive film in order to reduce background signal and prevent preferred particle orientations. Holey carbon support films are therefore popular for single-particle cryo-EM studies. Different holey carbon support films are available, such as regular perforated Quantifoil (Quantifoil Micro Tools GmbH, Jena, Germany) and C-flat (Protochips, Rayleigh, USA), as well as carbon films with holes of irregular size (*e.g.* lacey carbon film). It has been proposed (Brilot *et al.*, 2012 ▶) that differential behavior of carbon *versus* ice upon beam-illumination could cause a drum motion of the ice layer that leads to particle rotation and translation, and that varying dose rates and support-film hole size has an effect on the extent of these movements. Like us, Brilot *et al.* (2012 ▶) observed a gradual decrease of the amplitude of the beam-induced motions when the sample received more dose. Lower dose rates and smaller hole sizes appeared to reduce the beam-induced motions but it was impossible to avoid the motions entirely (Brilot *et al.*, 2012 ▶). Our attempts to investigate beam-induced motions as a function of variables such as beam *versus* hole size were hampered by the poor reproducibility of the observed motions for different grids used on different microscope sessions. This irreproducible character resembles our observations for room-temperature resin-embedded samples when charging compromised the image quality. Sections prepared from the same sample and supported by identically prepared grids and support films sometimes give variable susceptibility to charging, which, in general, is best overcome by the use of a thicker layer of carbon support.

Non-rigid registration based on gold fiducial markers can help to account for beam-induced movements observed in single-particle cryo-EM exposure series. The procedure, implemented in *Matlab* (http://www.mathworks.com/), takes a few seconds per image to run, and is available upon request. For the low doses used here, gold particles appear to move concurrent with the macromolecules within small patches of the imaged region. Correction based upon tracked beam-induced movements of gold particles thus seems to help, for the doses used, to improve the contrast and resolution of the examined macromolecule. Our observations resemble the findings of Brilot *et al.* (2012 ▶) that single-particle motion is correlated within patches of 0.3–0.5 µm. By fractionating the dose over a large number of frames, a computational correction can be applied to recover sharper images with higher contrast compared with the collection of single images with the same total dose. A natural neighbor scheme was employed and was combined with an accurate and robust tracking of gold fiducial markers. The latter depends on whether the fractioned dose is still sufficient to detect a significant fraction of the gold fiducials of a certain size at a certain magnification. The described procedures only aim to account for beam-induced movements, which is one of the consequences of radiation damage. Corrections for radiolytic effects such as bond breakage within the macromolecule of interest cannot be made with such schemes. Application of the non-rigid registration schemes will result in a dose-dependent data series that can be segmented into different subsets for different purposes (Diederichs *et al.*, 2003 ▶; Ravelli *et al.*, 2005 ▶; de Sanctis & Nanao, 2012 ▶). For example, summed images will improve particle picking as well as particle orientation determination, whereas the first image can be used for the calculation of the final three-dimensional reconstruction. Further studies should demonstrate whether or not non-rigid registration would also be compatible with the calculations of high-resolution three-dimensional reconstructions. In such cases it would be important to employ natural neighbor weighting schemes that prevent discontinuities (singularities) in the derivatives (Sugihara, 1999 ▶; Hiyoshi & Sugihara, 2004 ▶; Bobach *et al.*, 2006 ▶).

The robustness of the non-rigid registration scheme was examined by excluding small random subsets of the gold particles and using those as a test set to validate the correction based on the remaining gold particles (the ‘training set’). Typical residual errors on the gold particle positions of the test set were one to two pixels. Larger errors were found for test-set gold particles near the edge of the corrected area (outlined in yellow in Figs. 4 ▶ and 5 ▶).

Non-rigid registration might also be of relevance for electron tomography. Current popular tomography packages either cannot accommodate beam-induced movements (Inspect3D; FEI, USA) or use rigid registration schemes on partly overlapping subareas of the image (local alignments, IMOD; Mastronarde, 1997 ▶). Such schemes are not designed to deal with the kind of beam-induced movements described here and by Brilot *et al.* (2012 ▶). Non-rigid registration could become a useful new tool provided that caveats, such as reliable gold marker detection at high tilt angle and varying defoci, could be overcome. Systematic studies on untilted samples are necessary to develop priors on expected beam-induced movements which will help to validate gold particle tracks.

Rather than correcting for beam-induced movements computationally, it would be most desirable to reduce or remove such motions altogether. So far, however, it has remained unclear how to achieve such a goal without employing thick support films which compromise the signal-to-noise ratio. The combined use of an exposure series and computational corrections schemes will aid our understanding of beam-induced motions and thereby help to close the gap between experimental observations and theoretical predictions (Henderson, 1995 ▶).
